# Safety and efficacy of maraviroc (MVC) combined with multiple different therapeutic agents in highly treatment-experienced (TE) patients in Brazil

**DOI:** 10.1186/1758-2652-13-S4-P105

**Published:** 2010-11-08

**Authors:** JJD Furtado, JV Madruga, EL Bicudo, M Eira, MIB Lopes, EM Netto, MS de Oliveira, OHM Leite, AA Machado, U Tupinambas, JL de Andrade Neto, MPJ Lima, DB Wajsbrot, LM Cassoli

**Affiliations:** 1 Hospital Heliópolis, São Paulo, Brazil; 2 CRT - Centro de Referência e Treinamento DST/Aids, São Paulo, Brazil; 3 Unidade Mista n°1 - Centro de Referência n° 1 em DST AIDS (Hospital DIA), Asa Sul, Brazil; 4 Instituto de Infectologia Emílio Ribas, São Paulo, Brazil; 5 Divisão de Moléstias Infecciosas e Parasitárias do HC – FMUSP, São Paulo, Brazil; 6 UFBA - Hospital Universitário Prof. Edgard Santos, Salvador, Brazil; 7 Hospital Geral de Nova Iguaçu, Nova Iguaçu, Brazil; 8 Faculdade de Medicina do ABC, Santo André, Brazil; 9 Hospital de Clínicas da Faculdade de Medicina de Ribeirão Preto - USP - Unidade Especial de Tratamento de Doenças Infecciosas, Ribeirão Preto, Brazil; 10 Centro de Treinamento e Referência em Doenças Infecciosas e Parasitárias-Universidade Federal de Minas Gerais e Prefeitura, de Belo Horizonte, Brazil; 11 Pontifícia Universidade Católica do Paraná, Curitiba, Brazil; 12 Hospital e Maternidade Celso Pierro da Pontifícia Universidade Católica de Campinas - PUC-Campinas, Campinas, Brazil;; 13 Pfizer Inc, New York, USA; 14 Pfizer Brazil, São Paulo, Brazil

## Background

MVC is the first-in-class CCR5 antagonist approved for use in treatment of CCR5-tropic (R5) HIV-1 infection; however, there is limited experience with MVC in regimens containing newer PIs and other new agents. This open-label 96-week multi-center study evaluates MVC in a variety of regimens to obtain additional safety and efficacy data in TE patients with limited options due to intolerance or resistance in Brazil.

## Methods

Adult TE patients with R5 HIV-1 only (HIV-1 RNA >1000 cp/mL) received MVC 150-600mg (based on concomitant ARV) twice daily, combined with optimized background therapy (OBT). Every 12 weeks, safety parameters (primary endpoint), HIV RNA, and CD4 counts were assessed; we report data at 48 weeks.

## Results

Of treated 206 patients, 70% were male (mean age 43 yrs) and 80% Caucasian; 2.9% and 5.8% were seropositive for HBV or HCV infection, respectively. Median baseline HIV-1 RNA and CD4 counts were 4.9 log_10_ copies/mL and 185 cells/mm^3^, respectively. OBT comprised PI+NRTI (67%), PI+NRTI+NNRTI (7.8%), PI+NRTI+other (14.6%), or other regimens (10.2%). The most frequently used NRTIs were TDF (82%), 3TC (76%) and AZT (23%); the most common PIs (most boosted with RTV) were DRV (45%), LPV (41%) and ATV (15%); 16% received RAL. OBTs contained ≤1 drug (1% of patients), 2 drugs (7.3%), 3 drugs (24.3%), 4 drugs (35.4%), 5 drugs (23.8%), or ≥6 drugs (8.2%). Sixty-five patients (31.6%) discontinued; reasons included death (6 patients), adverse events (5), insufficient clinical response (36), lost to follow-up (4), or other (14). There were 238 treatment-related adverse events in 103 patients; 16 treatment-related serious adverse events in 9 subjects; 10 category C events, none treatment-related; and 2 of the 4 malignancies (Hodgkin's disease and intestinal T-cell lymphoma) were considered treatment-related. The most common grade 3/4 lab test abnormalities were GGT elevation (11% of patients), hyperbilirubinemia (11%) and serum amylase elevation (6%). Median CD4 count increased persistently through week 48 (Figure), with similar responses comparing quartiles of baseline CD4; the median increase from baseline at week 48 was 164.5 cells/mm^3^. HIV-1 RNA decreases were maintained regardless of baseline viral load; overall 41.1% of patients had HIV-1 RNA <400 copies/mL at week 48.

**Figure 1:**
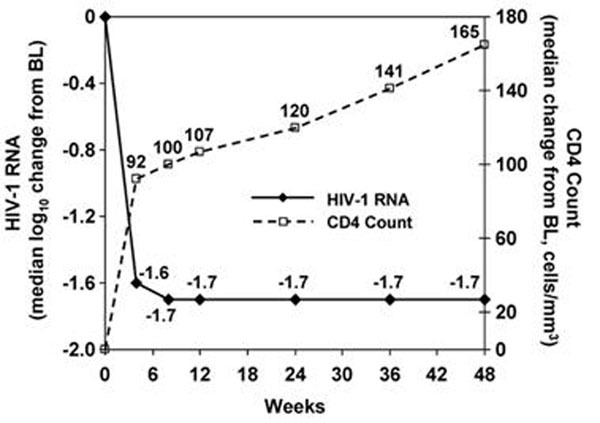


## Conclusions

In highly TE patients, regimens combining MVC with different agents from multiple classes were well tolerated and provided marked antiviral and immunologic responses.

